# Editors’ pick: Christmas is coming - time for chocolate to get ready for your Nobel Prize

**DOI:** 10.1186/2041-2223-3-26

**Published:** 2012-12-11

**Authors:** Manfred Kayser

**Affiliations:** 1 Department of Forensic Molecular Biology, Erasmus MC University Medical Centre Rotterdam, PO Box 2040, 3000 Rotterdam, CA, The Netherlands

## 

From early autumn, a number of shops, at least in Europe and presumably in many other places around the world too, are already preparing for Christmas by offering an abundant variety of chocolates, as is done every year. One almost has the joyous imagination that soon after the spring season all unsold Easter bunnies are called back into the chocolate factories and undergo reincarnation to stock the shops with the iconic chocolate Santa Claus of the winter season. Since an article was published in October 2012 by the *New England Journal of Medicine* I started to see chocolate, including bunnies and Santas, with somewhat different eyes. When I first heard about this paper, admittedly via the media, my first reaction was ‘this must be a joke’. After reading it, I think it is a funny paper demonstrating with an interesting example that correlations exist between many things, including those that may or may not have anything to do with each other (and no, I am not thinking of outcomes from genome-wide association studies on complex traits here).

So what is this all about? First of all, there are data suggesting that dietary flavonoids, such as flavanols present in cocoa, improve cognitive function [1]. From these findings, Franz H. Messerli, Director of the Hypertension Program of St Luke’s-Roosevelt Hospital and Columbia University, New York, hypothesized that such an effect may also exist at the population level [2]. In the absence of population data on cognitive function, Messerli arguably considered the number of Nobel laureates per capita as surrogate for a country’s citizen cognitive function. He found a strong and statistically significant positive linear correlation between chocolate consumption per capita and the number of Nobel laureates per 10 million people among the 23 countries studied (r=0.791, *P* <0.0001). Switzerland scored the highest, both regarding chocolate consumption (as may have been expected by the Swiss-origin author who is quoted in the article to consume chocolate on a regular daily basis, mostly Swiss-made chocolate) and also in the number of Nobel laureates. China scored the lowest with the lowest chocolate consumption among the 23 countries and no Nobel Prize listed (it appears that the Nobel Prize for peace was not considered in the analysis where the data collection deadline was in October 2011). Sweden appeared as an outlier with more than twice as many Nobel laureates than was expected given that country’s chocolate consumption; excluding Sweden from the analysis raised the correlation coefficient to 0.862. Two possible explanations for the outlier position of Sweden are offered by the author: either Swedes are especially sensitive to the assumed beneficial effect of chocolate on cognitive function, or the Stockholm-based Nobel Prize committee has a patriotic bias in their decisions of awarding Nobel Prizes. But can there indeed be a causal relationship between how much chocolate one eats and how extraordinary and groundbreaking one’s scientific achievements are? We cannot know without further studies, and Messerli suggested a prospective randomized trial to be carried out. Notably, he also points out the reverse scenario that a high cognitive function may stimulate chocolate consumption. Typically with these chicken-or-egg scenarios it will be difficult to elucidate what exactly is going on until the effects of chocolate ingredients, such as flavanols but also others, on human cognitive function are tested more thoroughly, for instance by experiments designed to explore the putative effect more directly.

With all this, it may be well worth the risk indulging in a big piece of chocolate, for instance the head of a Santa Claus (perhaps reincarnated from an Easter bunny), and quickly return to the lab or the PC for your next Nobel Prize-worthy experiment.

But most of all, have a Merry Christmas with lots of chocolate and some interesting reading such as (and hopefully not only) those populating the scientific literature.
